# Bilateral Ureteral Obstruction Secondary to Papillary Necrosis From Non-Steroidal Anti-Inflammatory Drug Use in an Adult Patient

**DOI:** 10.7759/cureus.16926

**Published:** 2021-08-05

**Authors:** Shannon J Leung, Theodore Cisu, Baruch M Grob

**Affiliations:** 1 Department of Urology, Virginia Commonwealth University School of Medicine, Richmond, USA; 2 Department of Urology, Virginia Commonwealth University Health System, Richmond, USA; 3 Department of Urology, Hunter Holmes McGuire Veterans Administration Medical Center, Richmond, USA

**Keywords:** ureteral obstruction, papillary necrosis, nsaid, non-steroid anti-inflammatory drug, renal papillary necrosis

## Abstract

Prolonged use of non-steroidal anti-inflammatory drugs (NSAIDs) is known to cause renal papillary necrosis and, in rare cases, can cause sloughing of renal papillae with subsequent ureteral obstruction. We report the first documented case of an adult patient presenting with bilateral ureteral obstructions, secondary to bilateral papillary necrosis from chronic NSAID use. He subsequently underwent bilateral ureteral stent placement with rapid recovery of renal function.

## Introduction

The use of non-steroidal anti-inflammatory drugs (NSAIDs), such as naproxen, is a known risk factor for the development of nephrotoxicity [1-3]. The inhibition of cyclooxygenase-1 (COX-1) and cyclooxygenase-2 (COX-2) by NSAIDs is the main physiological mechanism that can lead to renal dysfunction. COX-1 and COX-2 produce prostaglandins, which are responsible for regulating blood flow to the kidney. The inhibition of prostaglandin production by COX-1 and COX-2 causes unopposed renal vasoconstriction, leading to ischemia and acute renal papillary necrosis [3]. Although NSAIDs are largely considered safe medications, rare but life-threatening renal papillary necrosis can occur with prolonged use [2,3].

## Case presentation

A 34-year-old male presented to the emergency department for a 24-hour history of bilateral flank pain with oliguria and visible urinary sediment. He had a prior episode of anuria with urine sediment in the past while taking naproxen for his chronic headaches, which resolved after discontinuing naproxen and increasing fluid intake. One month prior to his presentation to the emergency department, he was prescribed naproxen 500 mg twice daily. Due to the patient's memory deficits from post-traumatic stress disorder (PTSD), he often ingested more than the prescribed amount according to the patient's wife but specific amounts were unknown.

**Figure 1:**
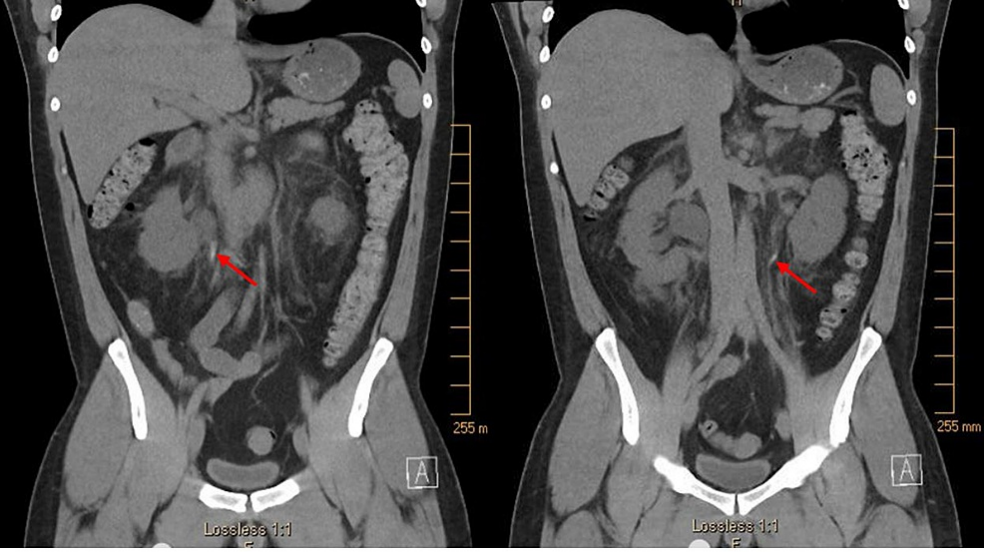
Abdominal and pelvic non-contrast CT revealing hyperdense material in bilateral proximal ureters.

**Figure 2:**
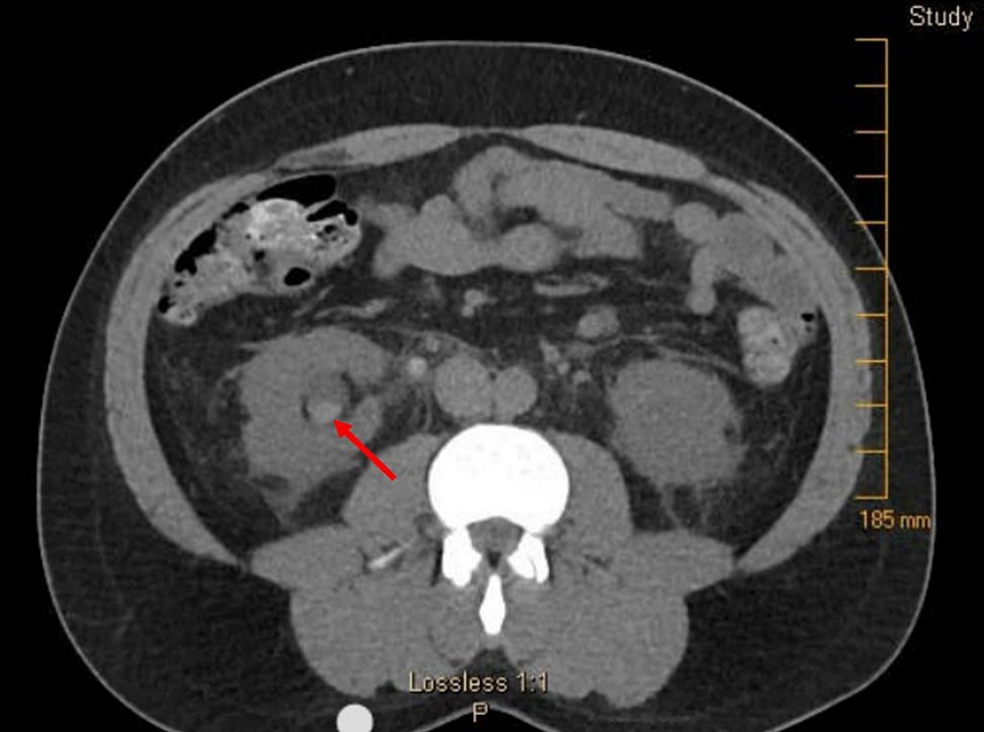
Abdominal and pelvic non-contrast CT revealing hyperdense material in right lower pole renal calyx.

**Figure 3:**
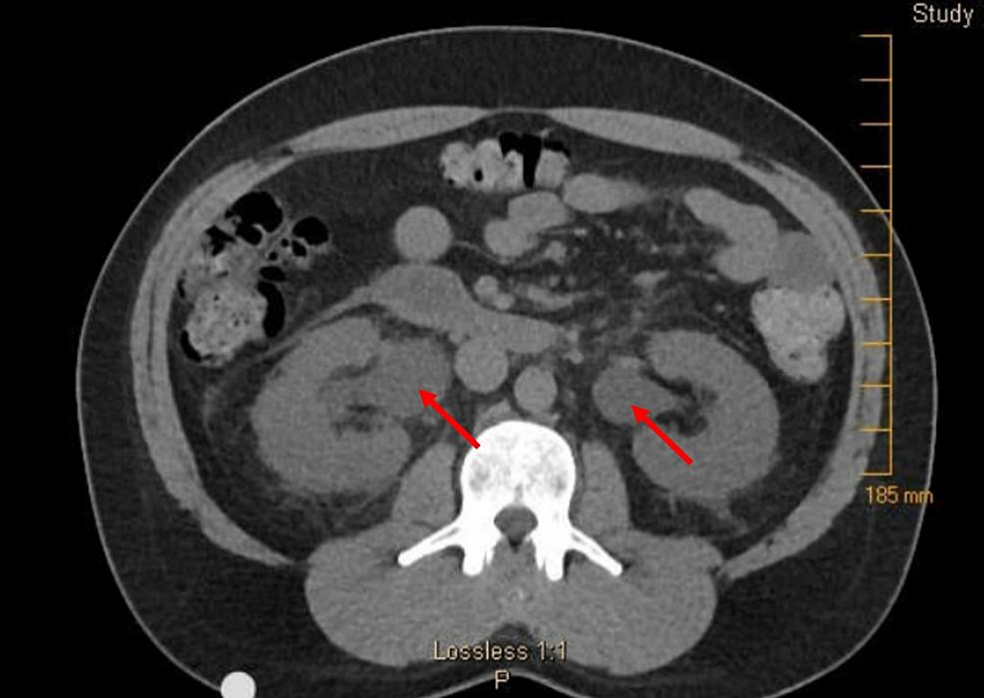
Abdominal and pelvic non-contrast CT revealing bilateral hydroureteronephrosis, suggesting obstruction.

The patient was anuric but hemodynamically stable, producing just 50 mL of urine despite >2 L of intravenous fluids in a 24-hour period. Laboratory investigations were notable for creatinine of 5.9 mg/dL, blood urea nitrogen (BUN) of 54 mg/dL, and glomerular filtration rate (GFR) of 11.72 mL/min/1.73 m^2^. Because the patient had no history of renal dysfunction other than the one prior transient episode of presumed naproxen-induced acute kidney injury, his renal function was thought to be normal at baseline. Complete blood count revealed leukocytosis of 14,000/mm^3^. Abdominal and pelvis non-contrast CT demonstrated hyperdense material in bilateral proximal ureters (Figure 1) and right lower pole renal calyx (Figure 2), with upstream bilateral hydroureteronephrosis (Figure 3). It was determined that the patient had bilateral ureteral obstruction, likely due to sloughing of renal papillae given the history of NSAID use. Because the patient reported he was comfortable and he remained hemodynamically stable, the decision was made to observe the patient overnight with IV hydration.

On patient assessment the following day, he was complaining of worsening bilateral flank pain and anuria. Labs revealed creatinine from 5.9 to 9.2 mg/dL, BUN from 54 to 72 mg/dL, GFR from 11.72 to 7.02 mL/min/1.73 m^2^, and leukocytosis from 14,000 to 15,000/mm^3^. Because of the patient’s worsening renal function, he was taken urgently for renal decompression. Cystoscopic examination in the operating room revealed a significant amount of matrix-like tissue in the bladder. Bilateral ureteral stents were placed without difficulty and proximal coils were confirmed in bilateral renal pelvises with fluoroscopy. Unfortunately, none of the matrix-like material was collected for histopathological analysis.

**Figure 4:**
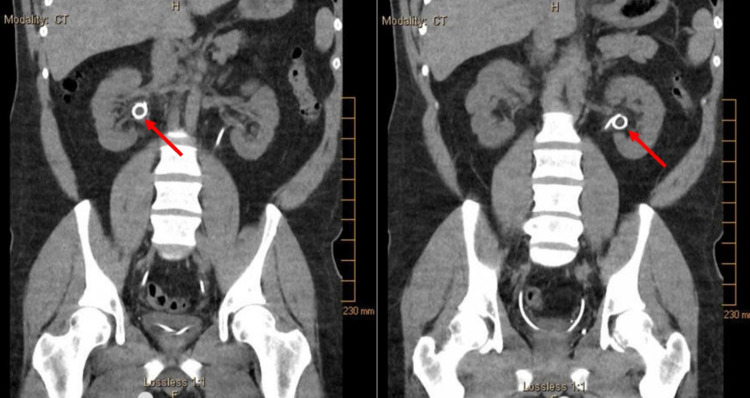
Abdominal and pelvic non-contrast CT with stent visualized in bilateral kidneys, ureteral obstructions, and hydronephrosis resolved.

The patient’s urine output promptly returned post-operatively with 24-hour urine output of 5.4 L; the high volume urine output was likely due to post-obstructive diuresis. The patient’s creatinine dramatically improved from a peak of 9.2 to 3.4 mg/dL on post-operative day two. The patient was advised not to take naproxen under any circumstances. Labs drawn nine days after his procedure revealed creatinine of 1.1 mg/dL, BUN of 17 mg/dL, and GFR of 81.44 mL/min/1.73 m^2^, confirming that his acute kidney injury had resolved. At his one-month post-operative outpatient appointment, repeat CT confirmed bilateral obstructions and hydroureteronephrosis had also resolved, and the stents were subsequently removed (Figure 4). The patient did not return for any further follow-up after stent removal, and therefore no other follow-up laboratory or imaging was available.

## Discussion

Although NSAIDs are generally considered safe, overuse can increase the risk for the development of renal papillary necrosis, as seen in this patient. Due to memory deficits, the patient was unable to quantify the amount of naproxen he was ingesting on a daily basis. Per the patient's wife, he was very likely taking more than his prescribed dose of 500 mg twice daily. The patient subsequently developed anuria, bilateral hydroureteronephrosis, and acute renal failure due to bilateral ureteral obstruction, likely from sloughed necrotic renal papilla. The presumed sloughed papilla can be seen on CT as the hyperdense material in the bilateral proximal ureters (Figure 1) and right lower pole calyx (Figure 2), which was subsequently visualized on cystoscopy after it progressed down the ureter and into the bladder.

NSAIDs as an inciting risk factor for renal papillary necrosis are well-documented; however, subsequent unilateral ureteral obstructions are uncommon and the development of bilateral ureteral obstructions is incredibly rare. Indeed, this is the first documented report of bilateral ureteral obstruction secondary to renal papillary necrosis from NSAID use in an adult patient. Most reports of ureteral obstruction from NSAID-induced renal papillary necrosis have been seen in pediatric cases [4-6], whereas the few documented adults' cases were secondary to ingestion of isolated COX-2 inhibitors [7,8] or a toxic household cleaner [9].

The pathophysiology of renal papillary necrosis with NSAID use is due to the drug’s inhibition of COX-1 and COX-2, which results in decreased production of prostaglandins and subsequent renal afferent arteriole vasoconstriction with renal papillary necrosis. The development of renal papillary necrosis in the setting of decreased blood flow can be explained by the anatomic vasculature of the renal papilla. Vascular bundles are widest in the outer medulla and gradually decrease in size, with only a few communicating or a single blood vessel in the papillary tip [8]. As a result, the renal papilla is most prone to developing ischemic necrosis.

The clinical presentation of renal papillary necrosis can often mimic other renal pathologic states, which leads to underdiagnosis or misdiagnosis. Flank pain and dysuria, which is present in about 50% of the cases, can result in renal papillary necrosis to be mistaken as renal stones [8]. It can also present similarly to urinary tract infections when the sloughed necrotic papilla causes obstruction, resulting in stagnation of urine in the calyces and becoming a nidus for infection [8].

A limitation of this case report is that the matrix-like tissue seen on cystoscopy was not sent for histopathological analysis to confirm the presence of renal papillary necrosis. However, given the patient's history of chronic NSAID use, acute kidney injury, and the presence of hyperdense material in the bilateral ureters on CT, it was determined that renal papillary necrosis was the most likely explanation for the patient's clinical presentation.

This patient’s progressively worsening renal function, with no prior history of renal disease, was a strong indicator that interventions were emergently needed to prevent irreversible damage. Bilateral ureteral stents were required to decompress the kidneys and to eliminate the obstruction caused by the presumed sloughed papillae tissue. In this case, the patient made a full recovery without evidence of permanent renal dysfunction. Without prompt surgical intervention, his acute renal dysfunction would likely have persisted and quickly worsened to the possible need for dialysis.

## Conclusions

In conclusion, severe acute kidney injury secondary to sloughed necrotic renal papillae causing ureteral obstructions is a rare but life-threatening condition that must be quickly identified and treated to prevent irreversible renal disease. Patients who chronically use NSAIDs must be counseled about the importance of avoiding overuse of these drugs to avoid the development of serious consequences, such as renal papillary necrosis. This case report highlights how early detection and intervention are crucial to prevent irreversible renal damage and possibly result in complete recovery.
